# Exometabolomic Analysis of Cross-Feeding Metabolites

**DOI:** 10.3390/metabo7040050

**Published:** 2017-10-04

**Authors:** Andrea Lubbe, Benjamin P. Bowen, Trent Northen

**Affiliations:** Lawrence Berkeley National Laboratory, Cyclotron Road, Berkeley 94720, CA, USA; alubbe@lbl.gov (A.L.); bpbowen@lbl.gov (B.P.B.)

**Keywords:** exometabolomics, bioprocessing, microbial consortia, cross-feeding, *Yarrowia lipolytica*, *Cellulomonas fimi*, lignocellulose

## Abstract

Microbial consortia have the potential to perform complex, industrially important tasks. The design of microbial consortia requires knowledge of the substrate preferences and metabolic outputs of each member, to allow understanding of potential interactions such as competition and beneficial metabolic exchange. Here, we used exometabolite profiling to follow the resource processing by a microbial co-culture of two biotechnologically relevant microbes, the bacterial cellulose degrader *Cellulomonas fimi*, and the oleaginous yeast *Yarrowia lipolytica*. We characterized the substrate preferences of the two strains on compounds typically found in lignocellulose hydrolysates. This allowed prediction that specific sugars resulting from hemicellulose polysaccharide degradation by *C. fimi* may serve as a cross-feeding metabolites to *Y. lipolytica* in co-culture. We also showed that products of ionic liquid-treated switchgrass lignocellulose degradation by *C. fimi* were channeled to *Y. lipolytica* in a co-culture. Additionally, we observed metabolites, such as shikimic acid accumulating in the co-culture supernatants, suggesting the potential for producing interesting co-products. Insights gained from characterizing the exometabolite profiles of individual and co-cultures of the two strains can help to refine this interaction, and guide strategies for making this an industrially viable co-culture to produce valuable products from lignocellulose material.

## 1. Introduction

Microorganisms exist in complex communities where different species with specialized phenotypes have different functional roles. There is great interest in harnessing the capabilities of microbial consortia to perform biotechnological and bioprocessing tasks [1]. Selecting and combining strains with specialized biochemical capabilities, potentially allows more complex tasks to be performed than any individual natural or engineered strain.

There are numerous examples where the division of labor between two or more microbial strains enables production of useful compounds from complex substrates [2]. A classic example of the combined bioprocessing capabilities of a co-culture is the mixed culture of *Trichoderma reseei* and *Saccharomyces cerevisae* on cellulose. The fungus degraded cellulose to glucose, and the yeast fermented glucose to ethanol [3]. Another recent example also employed *T. reseei* to degrade lignocellulose, cultured together with an engineered *E. coli* strain to produce isobutanol from the resulting sugar degradation products [4]. Bayer et al., [5] employed a cellulolytic bacterium *Actinotaleum fermentans* in co-culture with an engineered yeast to produce industrially important methyl halides from lignocellulose substrates. In this co-culture, acetate produced by *A. fermentans,* rather than small sugars, was consumed by the yeast in co-culture.

Besides the sequential utilization of cellulose and its degradation products in consortia, other interactions between consortium members can also be important, and must be considered in the design of a consortium. This may include competition for intermediates or products, and cooperative interactions such as beneficial metabolic exchange known as cross-feeding [2,6]. To truly understand such interactions, there is a need to track more than just the yield of the final desired product. Especially in the case of complex substrates such as lignocellulose, analytical methods capable of detecting a range of metabolites are needed, so that metabolic intermediates and products can be detected and tracked.

An approach that is well suited to this is exometabolomics, the analysis of small metabolite profiles in extracellular environments [7]. By studying metabolites consumed from or secreted into culture media, insights can be gained into the metabolic activity of individual microbial strains or communities [8,9]. Characterizing the substrate preference of individual microbial strains can reveal the potential for competition with other strains. For example, in the presence of a mixture of substrates, a particular microbial isolate may only consume a subset of these [10]. Characterizing metabolites produced by a strain can be used to identify potential cross-feeding compounds [11]. Additionally, the order in which substrates are consumed by a microbial strain may be tightly regulated, as is the case in catabolite repression systems [12]. Such knowledge about the substrate preferences, metabolic outputs, and order of consumption of metabolites by particular microbial strains can aid the design of a microbial consortium.

Here, we used exometabolite profiling to follow the resource processing by a microbial co-culture of two biotechnologically relevant microbes, *Cellulomonas fimi*, a bacterial cellulose and hemicellulose degrader, and *Yarrowia lipolytica*, an oleaginous yeast. *C. fimi* has been isolated from soil and compost heaps, and is a facultative anaerobic Actinomycete. Similar to other Cellulomonads, *C. fimi* degrades cellulose and hemicellulose by releasing polysaccharide-degrading enzymes into the extracellular environment [13]. *Y. lipolytica* is an ascomycete yeast that is able to accumulate a large fraction of its mass as neutral lipids. It is also genetically very tractable, which has made it an attractive model organism for the biotechnological production of high-value lipids and biofuel precursors [14]. Wild-type strains of *Y. lipolytica* are unable to utilize polysaccharides as a source of carbon. We hypothesized that the products of (hemi-) cellulose degradation by *C. fimi* could be channeled (cross-fed) to enhance the growth of *Y. lipolytica* and that exometabolomics will provide insights into isolate and co-culture metabolic dynamics.

## 2. Materials and Methods 

### 2.1. Chemicals

Ethanol-free chloroform, Pyridine, 98% methoxyamine hydrochloride, FAME marker standards and other reference standards were from Sigma Aldrich (St. Louis, MO, USA). N-methyl-N-trimethylsilytrifluoroacetamide (MSTFA) containing 1% trimethylchlorosilane (TMCS) was from Restek (Bellafonte, PA, USA). ^13^C-labelled standards were obtained from Omicron Biochemicals Inc. (South Bend, IN, USA).

### 2.2. Culture Methods

#### 2.2.1. Sugar Substrate Preference of *C. Fimi* and *Y. Lipolytica*

*Cellulomonas fimi* ATCC 484 and *Yarrowia lipolytica* W29 cultures were grown from glycerol stocks on R2A plates for three days at 30 °C. Plates were kept in 4 °C for up to three weeks. Basal Salt Medium (BSM) for culture of *C. fimi* [15] was prepared with the following ingredients (per L): 1 g NaNO_3_, 1 g K_2_HPO_4_, 0.5 g KCl, 0.5 g MgSO_4_, 0.5 g yeast extract. A single colony each of *C. fimi* and *Y. lipolytica* cells was used to inoculate 5 mL of BSM medium with 50 mmolar d-glucose in a 15 mL culture tube. Culture tubes were incubated overnight shaking (250 rpm) at 30 °C. The overnight cultures were grown to an OD (600 nm) of 0.975 and 1.131 for *C. fimi* and *Y. lipolytica*, respectively. Cultures (4 mL) were centrifuged (3000 × *g* for 8 min) and culture supernatant was removed. The cells were washed in blank BSM medium (without added sugar substrate), and re-suspended in 4 mL of blank medium. Three mL of culture medium in 15 mL culture tubes, each containing a different single sugar substrate (d-glucose, d-mannose, d-galactose, d-xylose, l-arabinose, or d-cellobiose at 50 mmolar), were inoculated with 30 µL of washed overnight cultures. Inoculated cultures were transferred to the wells of a sterile 96-well culture plate (250 µL each, n = 3). The plate was incubated in a Biotek Gen-5 plate reader at 30 °C with constant shaking (medium speed). OD measurements were recorded every 30 minutes for 30 hours to obtain growth curves.

#### 2.2.2. Substrate Preference of *C. Fimi* in a Mixture of Sugars

A single colony of *C. fimi* cells was used to inoculate 5 mL of BSM medium with 50 mmolar D-glucose in a 15 mL culture tube. Culture tubes were incubated overnight shaking (250 rpm) at 30 °C. The overnight culture was grown to an OD (600 nm) of 0.254. The culture (4 mL) was centrifuged (3000 × *g* for 8 min) and culture supernatant was removed. The cells were washed in blank BSM medium (without added sugar substrate), and re-suspended in 4 mL of blank medium. Fresh medium (8.5 mL of BSM with sugar mix—same sugars as in 4.2.1, 0.5 mmolar each) was inoculated with 10 µL of washed and re-suspended overnight culture. Inoculated culture (250 µL) was transferred to the wells of two sterile 96-well culture plates. The first plate was incubated at 30 °C in a Biotek Plate reader, constantly shaking and taking optical density readings (OD 600 nm) every 30 minutes to monitor growth of cells. The second plate was shaken (200 rpm) in an incubator at 30 °C. At various time-points, the contents of three wells from the second 96-well plate was collected, and centrifuged for 5 minutes at 10,000 × *g*. Ten µL of supernatant was collected, and added to 10 µL of ^13^C-labelled internal standard mix solution containing ^13^C6-glucose, ^13^C5-Xylose, ^13^C12-cellobiose and ^13^C-vanillic acid. After briefly mixing, 10 µL of this solution was transferred to a new microtube, and frozen at −80 °C. The first time-point was taken directly after inoculating the medium, and the rest were taken at two hour intervals from t = 14 h to 24 h. Once all samples were collected, frozen samples were lyophilized, and derivatized for GC-MS analysis.

#### 2.2.3. Co-culture of *C. Fimi* and *Y. Lipolytica* Cultured with Cellulose and Galactomannan

Experimental culture medium was prepared by adding 50 mg galactomannan and 50 mg cellulose to 50 mL BSM medium in 250 mL culture flasks. Polysaccharide substrates were autoclaved in the medium. Overnight cultures of *Y. lipolytica* and *C. fimi* were initiated in BSM medium with added glucose (1 g/L). Cultures were grown to OD of 1.080 (*Y. lipolytica*) and 0.154 (*C. fimi*), centrifuged, and washed in BSM with no added glucose and diluted ten times. Experimental culture media were inoculated with 0.1 mL of overnight cultures and 0.1 mL sterile water (for individual cultures), or 0.1 mL of each overnight culture for co-culture. Strains were cultured at 30 °C shaking at 200 rpm. At different time-points, starting immediately after inoculating, culture medium samples were collected, and dilutions were prepared for cell counts, performed on R2A agar plates. Differences in colony morphology and growth rates allowed colonies of *C. fimi* and *Y. lipolytica* to be easily distinguished and counted when both were present on the plates (Figure S1). Culture medium samples were also harvested, centrifuged, and 100 µL supernatant was collected for GC-MS analysis. Internal standard mixture (10 µL) consisting of ^13^C-labelled sugars and vanillic acid were added to samples prior to lyophilizing.

#### 2.2.4. Co-Culture of *C. Fimi* and *Y. Lipolytica* Cultured with Ionic-Liquids Treated Switchgrass

Minimal Salt Medium (MSM) components per liter were NH_4_SO_4_ (1 g), K_2_HPO_4_ (1 g), MgCl_2_ (0.25 g), NaCl (0.25 g). The medium was supplemented with a mineral mix (concentration in medium: 45.29 µM sodium citrate, 1.2 µM ZnSO_4_, 1.02 µM MnCl_2_, 17.77 µM Fe(II)SO_4_, 2 µM ammonium heptamolybdate, 1 µM CuSO_4_, 2 µM CoCl_2_, 0.338 µM sodium tungstate, 0.02 µM CaCl_2_), methionine (final concentration 10 mg/L), and vitamins (biotin and thiamine at 50 µg/L and 20 µg/L, respectively. The medium pH was adjusted to 7.0 before autoclaving. Experimental media were prepared by adding 5 mg of ionic liquid-pretreated switchgrass (IL-SG) lignocellulose to 5 mL culture medium in 15 mL borosilicate glass culture tubes. Tubes were capped with Kimax caps and autoclaved. Methionine and vitamin supplements were added after autoclaving. Overnight cultures of *Y. lipolytica* and *C. fimi* were initiated in BSM medium (with 0.5 g per L yeast extract) with added glucose (1 g/L). Cultures were grown to OD (600 nm) of 0.650 (*Y. lipolytica*) and 0.758 (*C. fimi*), centrifuged, and washed in blank medium (minimal salt medium with no added substrates or supplements) and diluted ten times. Experimental culture media were inoculated with 100 µL of overnight cultures and 100 µL sterile water (for individual cultures), or 100 µL of each overnight culture for co-cultures. Individual cultures of each strain and co-cultures of both strains were prepared in triplicate. Culture media were incubated at 30 °C shaking at 200 rpm. At different time-points, starting immediately after inoculating, culture medium samples were collected, and dilutions were prepared for cell counts, performed on R2A agar plates. On this medium, *C. fimi* produced bright yellow colonies, and *Y. lipoytica* produced cream-colored colonies, allowing easy counting even when both were present. The liquid culture medium was also harvested, centrifuged, and 100 µL supernatant was collected for GC-MS analysis. Internal standard mixture (10 µL consisting of ^13^C-labelled sugars and vanillic acid were added to samples prior to lyophilizing.

### 2.3. GC-MS Analysis

#### 2.3.1. Sample Preparation

Samples were derivatized for GC-MS analysis according to the method of Kind et al. [16]. Briefly, 10 µL of methoxyamine hydrochloride dissolved in pyridine (40 mg/mL) was added to each lyophilized sample, and shaken at 30 °C at maximum speed for 90 min using a thermomixer (Eppendorf). A mixture of retention time marker standards were prepared by dissolving fatty acid methyl esters (FAMEs) of different linear chain lengths in chloroform. The FAME mixture (20 µL) was added to 1 mL of N-methyl-N-trimethylsilytrifluoroacetamide (MSTFA) containing 1% trimethylchlorosilane (TMCS), and 90 µL of the FAMEs/MSTFA solution was added to each sample. Samples were shaken at 37 °C at maximum speed in a thermomixer for 30 min, and then transferred to and sealed in amber GC-MS sample vials containing glass inserts (Agilent). Extraction blanks were prepared following the above procedure but starting with empty Eppendorf tubes.

#### 2.3.2. Data Acquisition

Samples were analyzed using an Agilent 7890 gas chromatograph (Agilent Technologies, Santa Clara, CA) connected to an Agilent 5977 single quadrupole mass spectrometer, controlled by Agilent GC/MS MassHunter Acquisition software. Samples were injected using a Gerstel automatic liner exchange MPS system (Gerstel, Muehlheim, Germany) controlled by Maestro software. Sample injection volume was 2 µL, and the injector was operated in split-less mode. Samples were injected into the 50 °C injector port which was ramped to 270 °C in a 12 °C/s thermal gradient and held for 3 min. The gas chromatograph was fitted with a 30m long, 0.25mm ID Rtx5Sil-MS column (Restek, Bellefonte, PA, USA), 0.25 mm 5% diphenyl film with a 10 m integrated guard column. Initial oven temperature was set at 50 °C, and the over program was as follows: ramp at 5 °C/min to 65 °C, held for 0.2 min; ramp at 15 °C/ min to 80 °C, held for 0.2 min; ramp at 15 °C/min to 310 °C, hold for 12 min. The mass spectrometer transfer line and ion source temperature was 250 °C and 230 °C, respectively. Electron ionization was at 70 eV and mass spectra were acquired from 50 to 700 m/z at 8 spectra per second.

#### 2.3.3. Targeted Sugar Quantitation

Derivatized samples (0.5 µL) were injected on GC-MS. Sugar standard curves were prepared by analyzing standard mixes of the target sugars dissolved in BSM medium (without yeast extract) at different concentrations. Samples were prepared by adding 10 µL of ^13^C-labelled internal standard mix solution to 10 µL of each sample, and performing derivatization and sample preparation for GC-MS analysis as described above. Quantitative analysis was performed with MassHunter Quant software (Agilent). Metabolite peaks in samples and standards were normalized to ^13^C-labelled internal standards (C6-monosacchardies to ^13^C6-glucose, C5-monosaccharides to ^13^C5-xylose, and cellobiose to ^13^C12-cellobiose).

#### 2.3.4. GC-MS Untargeted Metabolite Analysis

Raw data were visually inspected using Agilent MassHunter Qualitative Analysis software (Agilent Technologies, Santa Clara, CA, USA). Agilent MassHunter Unknowns Analysis software v. B.07.00 (Agilent Technologies, Santa Clara, CA, USA) was used to perform spectral deconvolution, blank subtraction and library matching. Deconvoluted peaks were matched to entries in the Fiehn Metabolomics database (2013 version), using the FAME internal standards for retention time matching, and a mass spectral match factor of 75 or greater to determine putative hits. Deconvolution resulted in approximately 1000 features (peaks) per sample, of which about 20–40 per sample were “hits” matched to entries in the Fiehn database. Alignment of data and quality control was performed using Mass Profiler Professional software (Agilent Technologies). This yielded a smaller list of mass spectral features, each of which occurs in 100% of samples in at least one condition (one time-point in either a single culture or co-culture). Of these features, several dozen were putatively identified based on library matching. The list of metabolites was further manually curated by comparing putatively identified metabolites to reference standards, and cross-checking mass spectra with the NIST metabolite database. This resulted in a list of 62 and 42 metabolites with a high level of confidence in their assignments, in the first and second co-culture experiments, respectively.

## 3. Results and Discussion

### 3.1. Sugar Substrate Preference of C. Fimi and Y. Lipolytica

**Figure 1:**
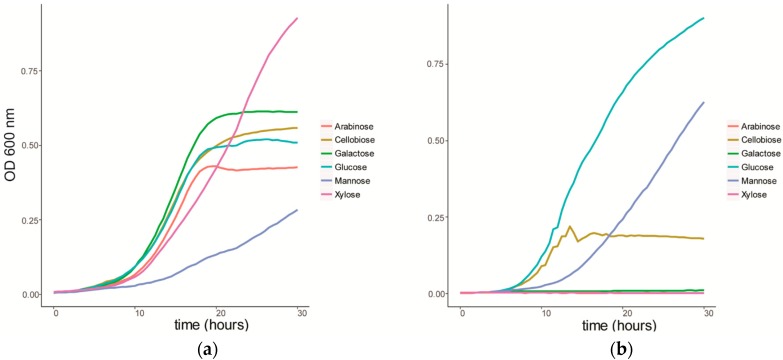
Growth curves of (**a**) *C. fimi* and (**b**) *Y. lipolytica* in Basal Salt Medium with individual sugars as carbon source.

We characterized the growth of *C. fimi* on individual sugars, representing typical products of cellulose and hemicellulose degradation (Figure 1a). *C. fimi* was cultured in Basal Salt Medium (BSM), a simple medium containing yeast extract and sodium nitrate and previously reported to support growth of this strain on polysaccharides [15], with different lignocellulose-related sugar monomers as carbon source. Growth of *C. fimi* was observed on all individual sugar substrates, with the fastest rate of growth observed with galactose, and the slowest with mannose. While the growth rate was slower for xylose than for most of the other sugars except mannose, the highest OD was reached when grown on this pentose sugar. *Y. lipolytica* was cultured on the same sugar substrates in BSM medium. Here significant growth was only observed with glucose and mannose as the main carbon sources (Figure 1b).

### 3.2. Substrate Preference of C. Fimi in a Mixture of Sugars

**Figure 2:**
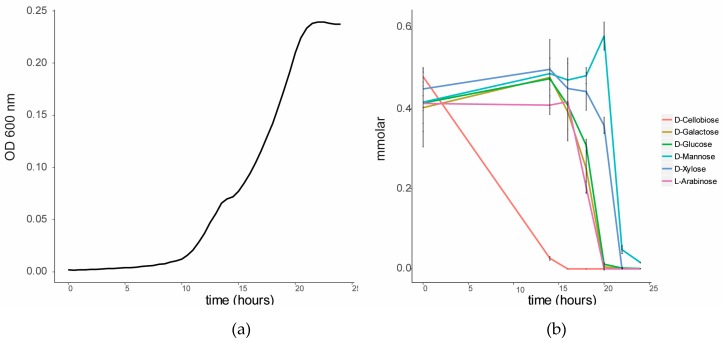
Sugar utilization by *C. fimi* in Basal Salt Medium with a mixture of sugars at 0.5 mmolar each. (**a**) Growth curve of *C. fimi* and (**b**) consumption of sugars over time.

To investigate the sugar substrate preference of *C. fimi*, this strain was cultured in BSM medium in the presence of a lignocellulose-related mixture of sugars. When cultured on this sugar mixture, a diauxic growth curve was observed (Figure 2). GC-MS analysis of the sugars in the culture supernatant over time showed that cellobiose was the first sugar to be consumed from the medium. Consumption of the other sugars only started once cellobiose was mostly depleted. Glucose, galactose and arabinose were consumed after cellobiose, and xylose and mannose were consumed last. The cellulolytic enzymes of members of the family Cellulomonadaceae are controlled by catabolite repression [17]. For example, the presence of glucose and cellobiose was reported to suppress degradation of carboxymethyl cellulose in *Cellulomonas uda* [18]. We hypothesized that in a co-culture with *Y. lipolytica*, the preferred consumption of cellobiose by *C. fimi* may make other sugars, such as mannose, available for consumption by the yeast.

### 3.3. Co-Culture of C. Fimi and Y. Lipolytica Cultured with Cellulose and Galactomannan

#### 3.3.1. Growth Curves on Cellulose Glucomannan Substrates

**Figure 3:**
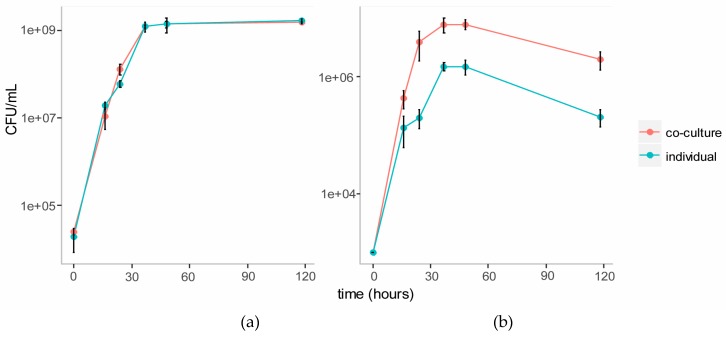
Growth curves of *C. fimi* (**a**) and *Y. lipolytica* (**b**) in individual and co-cultures in Basal Salt Medium with cellulose and galactomannan as carbon source.

We established a co-culture of the two strains in BSM medium with cellulose (composed of glucose monomers) and galactomannan (mainly composed of mannose and galactose monomers) as the carbon source. Individual cultures of each strain were also established in this medium as controls. Growth of each strain in the individual and co-cultures were tracked over five days using plate counts (Figure 3). The growth of *C. fimi* was similar in the individual versus the co-culture. Growth of *Y. lipolytica* was enhanced in the co-culture versus the individual culture.

#### 3.3.2. Targeted Sugar Analysis from Growth on Cellulose Glucomannan Substrates

**Figure 4:**
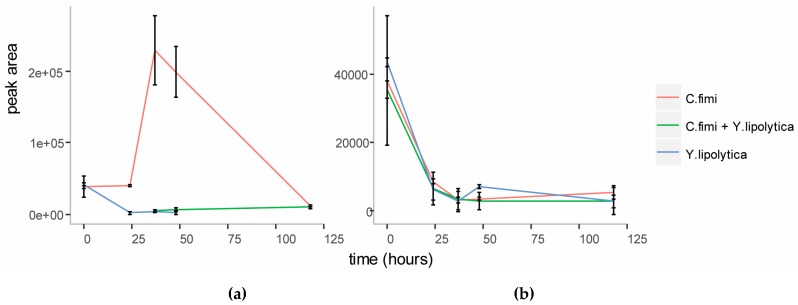
Relative quantitation of D-mannose (**a**) and D-glucose (**b**) in individual and co-culture supernatants in Basal Salt Medium with cellulose and galactomannan substrates.

To investigate whether mannose was responsible for the enhanced growth of *Y. lipolytica* in the co-culture, we first performed targeted analysis of sugars in the spent cellulose/galactomannan medium using GC-MS. This revealed that mannose accumulated in the medium at the end of logarithmic growth (Figure 4) in the individual *C. fimi* culture. After five days mannose was depleted from the medium again. This suggested that mannose at first accumulated in the medium as a result of polysaccharide degradation by *C. fimi,* but was consumed later. In the *Y. lipolytica* and *C. fimi* co-culture, mannose did not accumulate in the culture medium. Our interpretation is that mannose released into the medium by polysaccharide degradation, was consumed by *Y. lipolytica*, resulting in slightly enhanced growth. Galactose was not detected in the medium supernatants at any time point. Glucose, while detected at the start of the experiment in all cultures (possibly present as impurities in the polysaccharide substrates), did not accumulate in the medium at any of the time-points (Figure 4). This suggests that if glucose was released into the medium as a result of cellulose degradation, it was immediately consumed by *C. fimi*. Alternatively, it is possible that cellulose is not degraded all the way to glucose, but is broken down into glucose oligomers or the disaccharide cellobiose, and taken up by *C. fimi* in this form.

#### 3.3.3. Untargeted Metabolite Analysis on Cellulose Galactomannan Substrates

**Figure 5:**
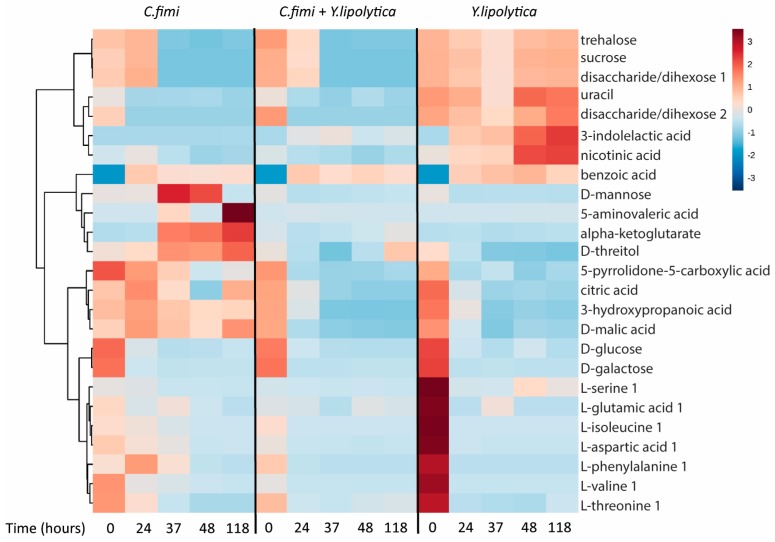
Clustered heatmap of metabolites in individual cultures and co-cultures of *Y. lipolytica* and *C. fimi* at different time points in Basal Salt Medium with cellulose and galactomannan as substrate.

To examine other metabolites beyond sugars that may be cross-fed between *C. fimi* and *Y. lipolytica* in the cellulose/galactomannan medium, the GC-MS data of the individual and co-culture supernatants were subjected to untargeted metabolite analysis. Metabolites identified at different time-points in the culture media of individual and co-cultures included sugars, organic acids, and amino acids (Table S1). To visualize patterns in the data, cluster analysis was performed to generate a heatmap, with the use of Metaboanalyst online tool suite (v.3.0, [19]). Data was mean-centered, Euclidean distance was used as distance measure, and Ward clustering algorithm was employed. The top 25 metabolites ranked as most significant by ANOVA (p < 5 × 10^−6^) were used to generate the heatmap, with the average peak area of each metabolite shown (Figure 5).

We see many examples of metabolites that accumulate in individual cultures, and are depleted in co-culture, suggesting cross-feeding, the reciprocal interspecies exchange of metabolites. Many of these are intracellular metabolites, e.g., alpha-ketoglutarate, uracil, serine, nicotinic acid, and threitol. Interestingly, we see the same behavior for mannose, which is almost certainly a product of galactomannan degradation. There is an increase and eventual consumption of mannose in the *C. fimi* culture, in contrast to the *Y. lipolytica* culture and co-culture. This, taken together with the data presented in Fig. 4, suggests that *C. fimi* secretes exogenous enzymes that produce mannose, benefiting *Y. lipolytica* which is known to utilize this sugar. *Y. lipolytica* is also able to utilize organic acids and sugar alcohols as carbon source [20]. It is therefore likely that the enhanced growth of *Y. lipolytica* observed in the co-culture was due to cross-feeding of both intracellular metabolites and extracellularly derived metabolic products. Reciprocally, metabolites such as nicotinic acid and 3-indolelactic acid accumulate in *Y. lipolytica* media are depleted in co-culture, though these do not show the same dramatic increase on *C. fimi* growth.

### 3.4. Co-Culture of C. Fimi and Y. Lipolytica Cultured With Ionic Liquid Treated Switchgrass

To determine whether similar interactions could be established with a complex lignocellulose substrate, co-cultures of *C. fimi* and *Y. lipolytica* were established with ionic-liquid treated switchgrass as the carbon substrate. Switchgrass is a non-food crop that has received much attention as a renewable feedstock for energy and other applications [21]. Ionic liquid treatment is a pretreatment method that aids deconstruction of recalcitrant lignocellulose, making polysaccharides accessible to enzymatic degradation [22]. The aim was to determine whether *C. fimi* could utilize this substrate, and release metabolites into the medium to cross-feed *Y. lipolytica,* resulting in increased growth as compared to when grown in isolation. After glucose, the main component of cellulose, the major sugar components of switchgrass lignocellulose are xylose and arabinose [21]. Since neither of these pentose sugars were preferred substrates of *Y. lipolytica* in the individual sugar experiments, we hypothesized that metabolites other than sugars may be responsible for cross-feeding in this co-culture.

#### 3.4.1. Growth Curves on Ionic Liquid Treated Switchgrass

**Figure 6:**
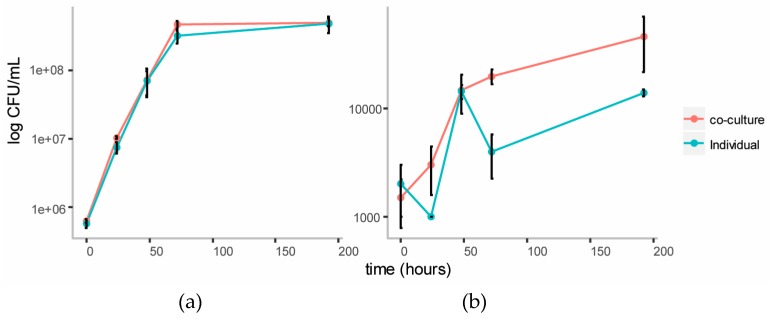
Growth curves of *C. fimi* (**a**) and *Y. lipolytica* (**b**) in individual and co-cultures in Minimal Salt Medium with Ionic Liquid-treated switchgrass lignocellulose as carbon source.

In order to observe cross-feeding metabolites responsible for the growth of *Y. lipolytica* more clearly, the Basal Salt Medium was simplified by omitting yeast extract from the medium in this experiment. The medium was supplemented with the vitamins biotin and thiamine, reported to be essential for growth of members of genus *Cellulomonas* [23]. Methionine was also included as a supplement, as this amino acid is reported to play an essential role in chemotaxis of *C. fimi* to sugars [17]. Ammonium in the form of NH_4_SO_4_ was included as the source of nitrogen, to allow for better growth of *Y. lipolytica*, which is unable to assimilate nitrate [20]. In this new simpler medium (Minimal Salt Medium), individual cultures of *C. fimi* grew slower, reaching stationary phase after about four days (Figure 6). Compared to the individual culture, cell counts of *C. fimi* in the co-culture were higher during logarithmic growth phase. Growth of *Y. lipolytica* was enhanced in the presence of *C. fimi* as compared to the individual culture. At the 72 hour time-point cell counts of *Y. lipolytica* in the co-culture were significantly higher, *t*(4) = 7.73, *p* = 2 × 10^−3^, than in the individual culture.

#### 3.4.2. Untargeted Metabolite Analysis from Ionic Liquid-Treated Switchgrass

**Figure 7:**
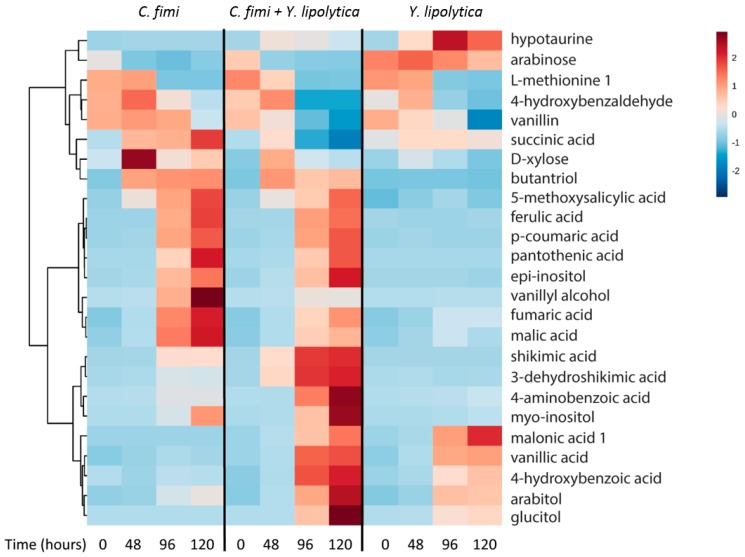
Clustered heatmap of metabolites in individual cultures and co-cultures of *Y. lipolytica* and *C. fimi* at different time points in defined medium with ionic liquid-treated switchgrass lignocellulose as substrate.

GC-MS data of the individual and co-culture supernatants were subjected to untargeted metabolite analysis as described above to investigate other potential cross-feeding metabolites. Metabolites identified at different time-points in the culture media of individual and co-cultures included organic acids, sugars, sugar alcohols, and several phenolic compounds (Table S2). Hierarchical cluster analysis was performed on the identified metabolites, and results of 25 metabolites with significant differences between cultures and time-points (*p* < 1 × 10^−3^) are presented in Figure 7.

The pentose sugar xylose was detected in the *C. fimi* culture medium at the 48 hour time-point, reaching a maximum at this time and decreasing thereafter. Xylose was relatively lower at these time-points in the co-culture, indicating that *Y. lipolytica* may have consumed it. Even though xylose was not depleted in the individual sugar culture experiments of *Y. lipolytica*, there have been reports of this strain utilizing xylose in certain conditions [24]. Arabinose was present in the culture supernatants at the start of the experiment, and was consumed by *C. fimi* but not *Y. lipolytica*. Once *C. fimi* reached stationary phase, metabolites such as ferulic acid, p-coumaric acid, and vanillyl alcohol were detected in the culture medium. The vitamin pantothenic acid, isomers of inositol, and the organic acids malic acid and fumaric acid also displayed this pattern. Of these metabolites, several were detected at lower abundances when *Y. lipolytica* was also present in the co-culture. These included vanillyl alcohol, fumaric acid, and malic acid.

Vanillin was detected at the t=0 time-point in all samples, suggesting it was present in the original substrate. Vanillin decreased over time in the *Y. lipolytica* individual culture, as well as the co-culture. Vanillic acid, not detected in the first time point, increased over time in the *Y. lipolytica* individual culture and co-culture. Relative amounts of hypotaurine increased in the *Y. lipolytica* culture medium over time. A number of metabolites accumulated only in the co-culture medium supernatants. These included shikimic acid, dehydroshikimic acid, 4-aminobenzoic acid, and glucitol.

Overall, like the cellulose and galactomannan experiment, we detected many metabolites that are abundant in the isolate spent media that are depleted in co-culture, suggesting cross-feeding. For example, malate and succinate accumulate in *C. fimi* mono-culture, but are significantly depleted in co-culture (*p* = 2 × 10^−8^ and *p* = 2 × 10^−4^ for malate and succinate at the 120 hour time-point, respectively). Hypotaurine was abundant in the *Y. lipolytica* mono-culture, and significantly lower in the co-culture at the last time-point (*p* = 1.32 × 10^−8^). Again, we also observe examples of biomass-derived metabolites such as vanillyl alcohol that are elevated in *C. fimi* mono-culture vs. co-culture (*p* = 6 × 10^−22^). Thus, it seems reasonable to consider cross-feeding more generally in terms of exchange of intracellular and extracellular metabolic products.

It was particularly interesting to observe accumulation of several metabolites in higher levels in co-cultures than mono-cultures, for example shikimic acid and deconstruction product, 3-dehydroxyshikimic acid, (Figure 7) suggesting a metabolic interaction between the two strains driving accumulation, for example *Y. lipolytica* inhibiting *C. fimi* uptake. This is exciting because shikimic acid is a valuable precursor, and is the starting material for synthesis of oseltamivir (Tamiflu) [25] and would be a useful co-product. We also observe several metabolites, especially the phenolic compounds ferulic- and coumaric acid, that accumulated in the culture medium as a result of lignocellulose degradation. While neither of the strains could utilize these compounds, they could potentially be substrates for additional strains in a more complex microbial consortium. The addition of such phenolic compound-degrading strains could further channel carbon towards the oleaginous yeast.

Co-culture with *C. fimi* was found to increase the growth of *Y. lipolytica* on polymeric substrates, and we observe the depletion of diverse metabolites in co-culture vs. monocultures, which we attribute to cross-feeding. However, there are alternative explanations, for example, interspecific interactions may alter substrate preferences in co-culture vs. mono-culture. Reductionist experiments using mutants would be required to further unravel exactly what occurs in co-cultures. While this is beyond the scope of this work, the metabolomics methods and insights into potentially cross-feeding metabolites described provide important tools and insights to guide additional studies.

## 4. Conclusions

We used exometabolomics to characterize the substrate preferences of two microbial strains, both as isolates and in co-culture, to identify potential cross-feeding metabolites. Efforts focused on substrates typically found in lignocellulose hydrolysates, to allow predictions of resource partitioning between the two microbes when cultured on polysaccharides and ionic liquid-treated switchgrass lignocellulose. We found that *C. fimi* grew on these substrates, and *Y. lipolytica* consumed products of the lignocellulose degradation. In the more defined medium with ionic liquid-treated lignocellulose as substrate, a number of metabolites released by *Y. lipolytica* into the culture medium appeared to be consumed by *C. fimi*. We also observe the accumulation of metabolites, such as shikimic acid, that may be valuable co-products, or be used to inform metabolic engineering strategies for broadening the substrate range of *Y. lipolytica*. Overall, this experimental approach shows promise for exploring the biochemical capabilities of other potentially useful organisms. Insights gained from characterizing the exometabolite profiles of individual and co-cultures of strains can help to refine interactions and guide strategies for making this an industrially viable co-culture to produce valuable products from lignocellulose material.

## Supplementary Materials

The following are available online at www.mdpi.com/2218-1989/7/4/50/s1, Table S1: Metabolites detected in individual and co-cultures of *C. fimi* and *Y. lipolytica* grown on cellulose and galactomannan, Table S2: Metabolites detected in individual and co-cultures of *C. fimi* and *Y. lipolytica* grown on ionic liquid-treated switchgrass lignocellulose. Figure S1: Petri dish with R2A medium with colonies of *C. fimi* (small, yellow, circular, entire) and *Y. lipolytica* (large, cream-colored, circular) after four days of growth at 30 °C.
