# Supplementary material for: Exometabolomic Analysis of Cross-Feeding Metabolites
Source: Metabolites. 2017 Oct 4;7(4):50. doi: 10.3390/metabo7040050 (PMC5746730; doi:10.3390/metabo7040050)
Supplement: Supplementary file 1 [file metabolites-07-00050-s001.zip › Figure S1.docx]

**Supplementary Materials: Exometabolomic analysis of cross-feeding metabolites**

# Andrea Lubbe ^1^and Trent Northen ^1,^*

| 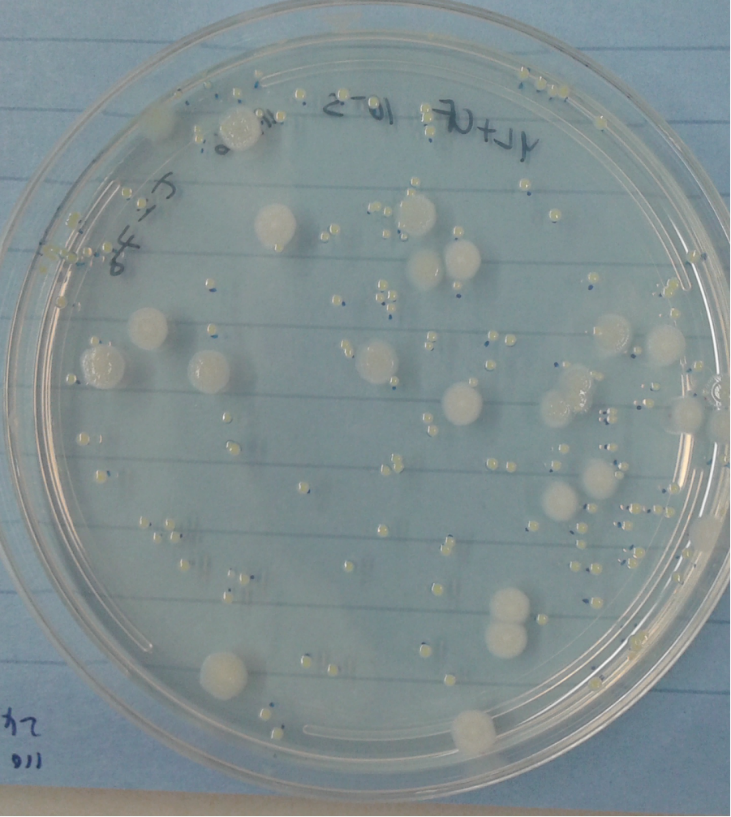 |  |
| --- | --- |

**Figure S1.** Petri dish with R2A medium with colonies of *C. fimi* (small, yellow, circular, entire) and *Y. lipolytica* (large, cream-colored, cicular) after four days of growth at 30°C.
